# Analysis of the Incidence and Survival of Gastric Cancer Based on the Lauren Classification: A Large Population-Based Study Using SEER

**DOI:** 10.3389/fonc.2020.01212

**Published:** 2020-08-03

**Authors:** Chao-Tao Tang, Ling Zeng, Jing Yang, Chunyan Zeng, Youxiang Chen

**Affiliations:** Department of Gastroenterology, the First Affiliated Hospital of Nanchang University, Nanchang, China

**Keywords:** gastric cancer, Lauren classification, SEER, incidence, survival

## Abstract

**Background:** Limited evidence exists on the incidence of gastric cancer (GC), and contradictory results exist for the prognosis of GC based on the Lauren classification. We analyzed the incidence and survival of GC based on the Lauren classification.

**Methods:** The Surveillance, Epidemiology, and End Results (SEER) database from 1975 through 2015 was used to identify all patients with surgically resected, histologically diagnosed intestinal or diffused-type GC. Propensity score matching was used to analyze the association between the Lauren classification type and prognosis.

**Results:** The trend of total GC incidence showed an obvious decrease (APC = −1.51, 95% CI: −2.31 to −1.01) as well as that of the intestinal type (APC = −1.43, 95% CI: −2.01 to −1.12). However, we found that the relative incidence of the diffused type was increased (APC = 0.6, 95% CI: 0.41–0.82). The trend of the total incidence of GC (APC = −1.31, 95% CI: −1.91 to −1.03) and that of the intestinal type (APC = −1.11, 95% CI: −1.53 to −0.98) was decreased in 40–49-year-olds, but that of the diffused type was increased (APC = 1.5, 95% CI: 1.2–1.72). We found that trends in GC incidence exhibited a similar pattern in the regional and distant stages and showed a decrease from 1975 through 2015. However, the incidence rate of the local stage was increased, with an APC of 0.5 (95% CI: 0.3–0.7). We identified 15,989 GC cases from the SEER database, including 13,852 intestinal-type and 2,138 diffused-type cases. The 1,336 intestinal-type cases were matched with 1,336 diffused-type cases using propensity score matching (PSM), and patients with the diffused type had a better prognosis than patients with the intestinal type (HR = 0.56, 95% CI: 0.45–0.78). However, we found that patients with diffused-type GC had worse survival than patients with intestinal-type GC in the cohort from Renji Hospital (*P* < 0.001).

**Conclusion:** The total incidence of GC and that of the intestinal-type GC decreased, but the incidence of diffused-type GC increased in 40–49-year-olds. Diffused types of GCs may have a different prognosis compared to intestinal-type GCs in different patient cohorts. Nevertheless, these results should be interpreted with caution in assessing the prognosis in combination with other factors.

## Introduction

Global cancer statistics reveal that gastric cancer (GC) is the fifth most common cancer, with more than 1,000,000 new cases diagnosed and an estimated 783,000 deaths in 2018; GC has a greater impact in Eastern Asian countries, such as Mongolia, Japan, and Korea, compared to Northern America and Europe (1). The primary cause of GC is chronic infection with *Helicobacter pylori* (Hp), which accounts for ~89% of cases that occur in the gastric antrum (2). Hp was defined as a class I carcinogen in 2009 (3). Other factors, including smoking, obesity, reduced physical activity, and dietary factors, enhance resistance to antibiotics, which leads to the failure of the eradication of Hp (4, 5). Some data showed that Hp eradication reduced the incidence of GC, especially in Asian countries, including China and Japan (6, 7). The risk of developing GC in patients who eradicated Hp was 34% lower than that in patients without Hp eradication (6). The epidemiological data of gastric cancer varies greatly due to the implementation of early screening and surveillance of GC using endoscopy and biomarkers (8, 9). For example, endoscopic screening greatly aids in the detection of early GC and prevents the progression of GC by removing precursor lesions, such as high-grade or low-grade dysplasia (9). Despite this background, there is little evidence on whether the incidence of GC and other epidemiological characteristics were altered over the past several decades.

The Lauren classification was first proposed in 1965, and it is widely accepted by clinicians because of the connection between histopathology and other clinical indicators (10). Intestinal-type GCs primarily originate from precursor lesions caused by Hp-induced chronic inflammation (11). In contrast, diffused-type GCs are induced by active inflammation (12). The classification divides GC into three types: intestinal, diffused, and mixed types. Intestinal-type GCs are prevalent in elderly patients, and diffused-type GCs tend to occur in younger patients (10). As for the genomic analysis, 40% of diffused-type GC was associated with CHD1 (chromodomain-helicase DNA-binding protein 1) loss; additionally, diffused-type GC was inclined to have mutant p53 and oncoprotein Her2, which induced diffused-type GC to be more aggressive type (13). However, other genes that were overexpressed in intestinal-type GC were also associated with poorer survival such as Claudin 6 (CLDN6) (14). There was little literature to investigate the difference of prognosis for patients with intestinal-type and diffused-type GC. Some studies showed that patients with intestinal-type GC had a better 5-year overall survival than diffused-type GC patients (15). However, a propensity score matching study based on the SEER database found that the rates of 5-year cancer-specific survival in patients with the intestinal-type and diffused-type early GC were comparable (16). It is controversial whether the survival of patients between intestinal and diffused-type GC was different.

Therefore, in order to investigate the alteration of incidence of intestinal- and diffused-type GC, we performed a comprehensive analysis using SEER database. Besides, regarding the disparities between intestinal- and diffused-type GC, we performed PSM to compare their survival.

## Methods

### Incidence

We designed and performed our study as described previously (17). Briefly, the SEER program, which covers almost 30% of the population in the United States, was used to assess trends in gastric cancer incidence by pathological type, including the intestinal type (M8140, M8211, M8010, and M8144) and diffused type (M8145, M8490, and M8142) (16), and stage according to SEER historical staging. We included all cases diagnosed in patients older than 20 years of age from 1975 to 2015 in the SEER program. SEER^*^Stat software (version 8.3.6) was used to analyze age-standardized incidence rates to the 2000 US standard population and assess annual percentage changes (APCs) since 1975. The incidence rates of the age groups were reported as the number of cases per 100,000 people.

### Patients

All patients with gastric cancer were retrieved from the SEER database. None of the patients provided informed consent because the SEER database is free for the public. Patients with GC were included in our study according to the following criteria: (1) patients aged more than 20 who were diagnosed with GC by positive histology from 1979 through 2015, (2) patients with histopathology of intestinal-type or diffused-type GC, and (3) patients with detailed information, including age, sex, race, pathological grade, regional node examination, regional node positive status, tumor size, T stage, N stage, and M stage.

### Clinicopathological Factors

The clinicopathological variables extracted from the SEER database in our study included age, race, sex, pathology grade, N stage, M stage, tumor size, T stage, and regional node ratio. The age of patients was divided into two groups, <60 and ≥60 years. Race was classified into three types: white, black, and other. Sex included male and female. Pathology grade was categorized as a well/moderately differentiated type and poorly differentiated/undifferentiated type. LNM was described as N1 (Yes), and N0 was negative. M1 (Yes) indicated positive. GC tumors were categorized into 4 size groups: ≤2, ≤3, ≤5, and >5 cm. The regional node ratio was determined as regional nodes positive and regional nodes examined, according to the results of the K-adaptive partitioning (KAPS) algorithm (18), and the cutoff was 0.56. Therefore, regional nodes examined was divided into two groups, ≤0.56 and >0.56. The primary observation indicators were overall survival (OS) and cancer-specific survival (CSS). CSS was defined as the length of time from either the date of diagnosis or the start of treatment for cancer to the date of death from cancer.

### Statistical Analysis

For the basic statistics, patients were divided into two groups according to intestinal type and diffused type, and Pearson's chi-squared test was used to investigate the associations with the categorical variables. With respect to CSS for patients, we performed survival curves using the survminer package in R software. We performed propensity score matching (PSM) because of the imbalance between the two groups to generate a new data set for analysis using the MatchIt package in R software. The value of the caliper was set as 0.05, and the effect was valued as the *P*-value. Balance was indicated when the *P*-value was >0.05 (19). The following detailed process was used. We initially calculated the propensity scores of each patient according to histology using a multivariate logistic regression model. We then matched patients between the two groups at a ratio of 1:1. Detailed information of all clinical factors is listed in **Table 2**. We analyzed the differences of all variables between the two groups using the chi-squared test. Finally, we examined the correlation between other factors and histological type using the univariate Cox regression model. A plot of cumulative events was also constructed. All statistical analyses were performed in R software (version 3.6.1, StataCorp LLC, College Station, Texas, USA). The survival curves was calculated using the “rms,” “survminer,” and “survival” packages in R software (Version 3.5.0), of which ggsurvplot function was used to perform the K–M survival curve. All packages used in our manuscript were obtained from the website https://www.r-project.org/, or the function of “help” in the R software was used. The chi-squared test was performed in SPSS (version 24.0). All results were considered to be statistically significant when the *P*-value was <0.05. Differences were considered statistically significant when the value of *P*-was <0.05.

## Results

### Incidence Between Intestinal-Type and Diffused-Type GC

**Figure 1:**
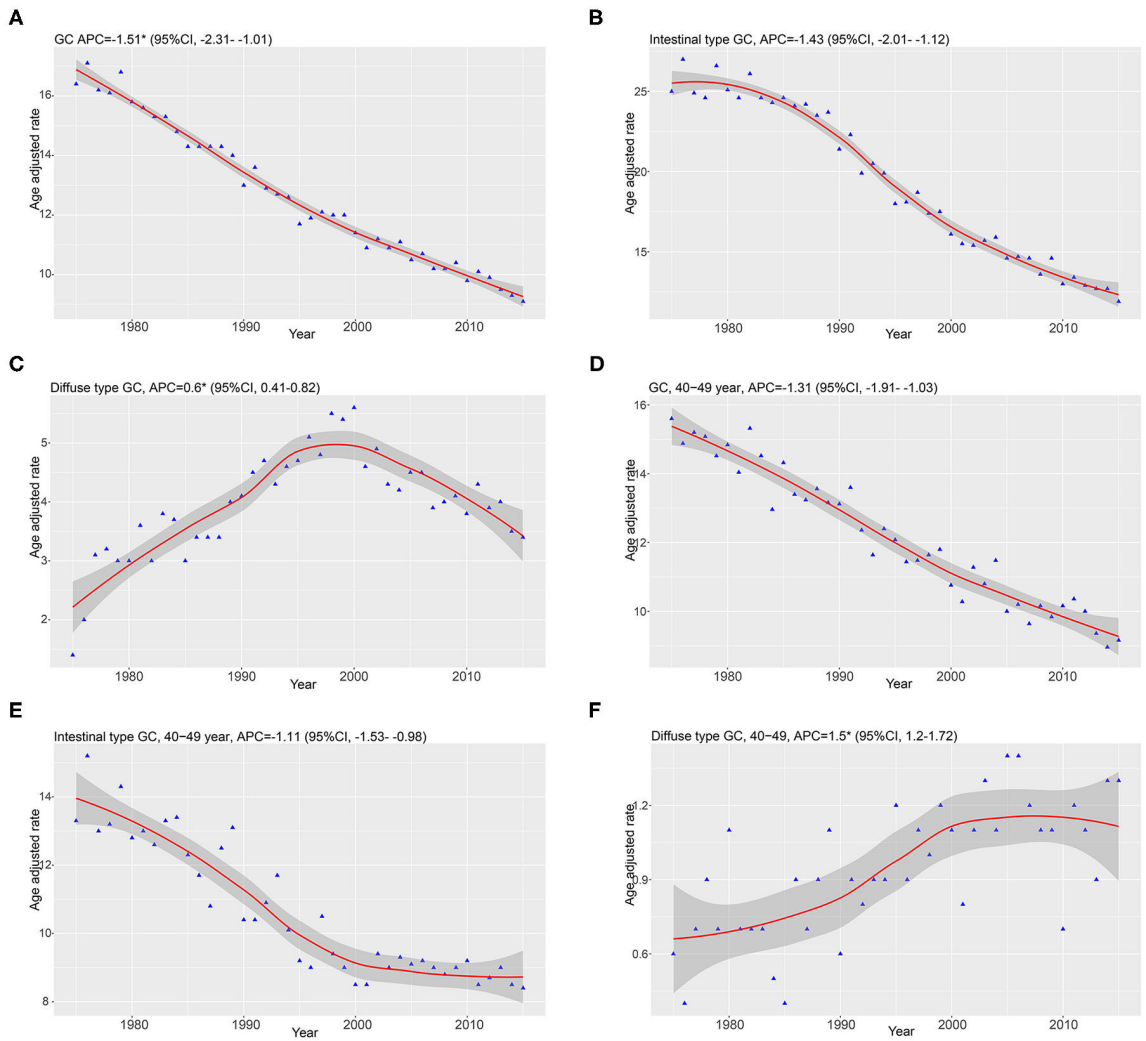
Data are from the Surveillance, Epidemiology, and End Results program and were age-standardized to the 2,000 US standard population. Smooth lines represent fitted piecewise–log linear trends. Annual percentage changes (APCs) with parametric confidence intervals were calculated. **(A–C)** Represented the trends of GC, diffused type, and intestinal type. **(D–F)** Showed the trends of GC, diffused type, and intestinal type diagnosed at 40–49 years of age.

**Figure 2:**
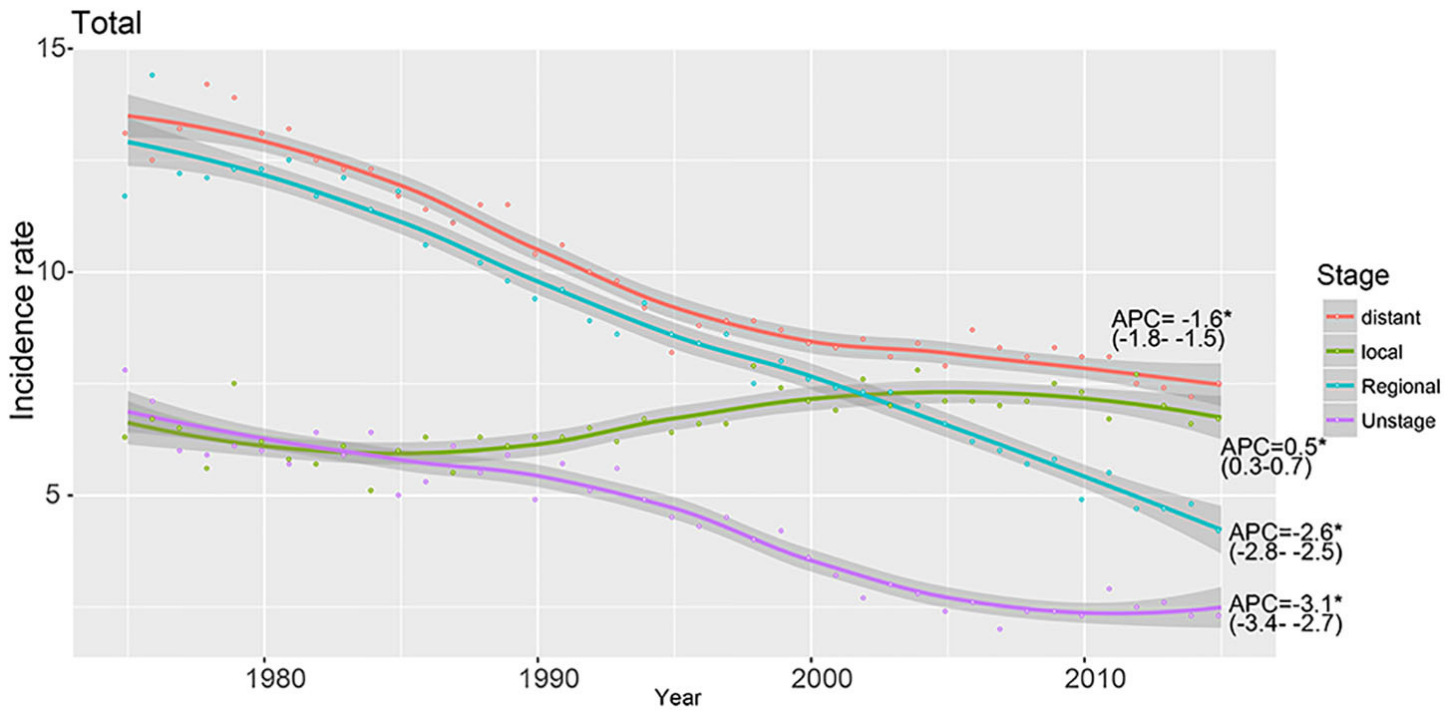
The incidence of GC at different stages is shown with APC.

Our study enrolled 15,989 GC cases who were diagnosed at age higher than 20 years from 1975 to 2015. There were 13,851 cases diagnosed as intestinal type and 2,138 cases diagnosed as diffused type. The total trends in GC incidence showed an obvious decrease (APC = −1.51, 95% CI: −2.31 to −1.01; Figure 1A). The incidence of the intestinal type decreased with an APC value of −1.43 (95% CI: −2.01 to −1.12; Figure 1B). However, in contrast to the total change, we found that the relative incidence of the diffused type was increased because the APC was 0.6 (95% CI: 0.41–0.82; Figure 1C). Notably, the incidence rate of diffused-type GC exhibited a significant decreasing trend from 2000 to 2015 and increased significantly from 1975 to 2000 (Figure 1C), with APC values of −1.2 (95% CI: −2.3 to −0.1) and 2.6 (95% CI: 1.8–3.1), respectively. We analyzed the trend of the incidence of GC in 40–49-year-olds. The results showed that incidence for GC (APC = −1.31, 95% CI: −1.91 to −1.03) and intestinal-type carcinoma (APC = −1.11, 95% CI: −1.53 to −0.98) decreased, but the incidence of diffused-type carcinoma increased (APC = 1.5, 95% CI: 1.2–1.72; Figures 1D–F). Our analysis of stage distribution found that the trends in GC incidence exhibited a similar pattern across regional and distant stages and unstaged cases (Figure 2), which were decreased from 1975 through 2015, with an APC of −2.6 (95% CI: −2.8 to −2.5), −1.6 (95% CI: −1.8 to −1.5), and −3.1 (95% CI: −3.4 to −2.7), respectively. The incidence rate of the local stage increased, with an APC of 0.5 (95% CI: 0.3–0.7).

### Survival Between Intestinal-Type and Diffused-Type GC

**Figure 3:**
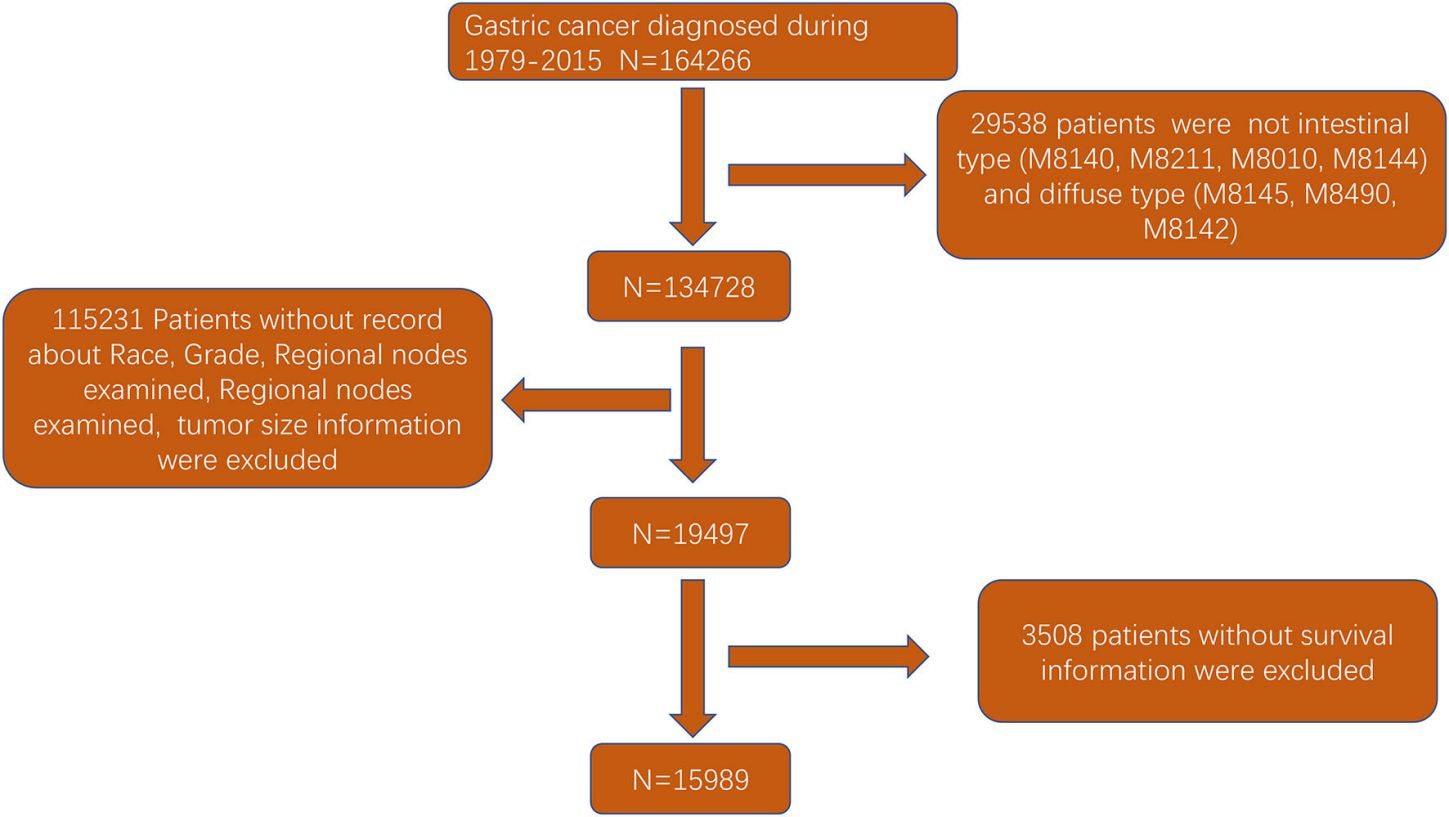
Flowchart of selection of patients with GC of intestinal type or diffused type using the SEER database.

**Figure 4:**
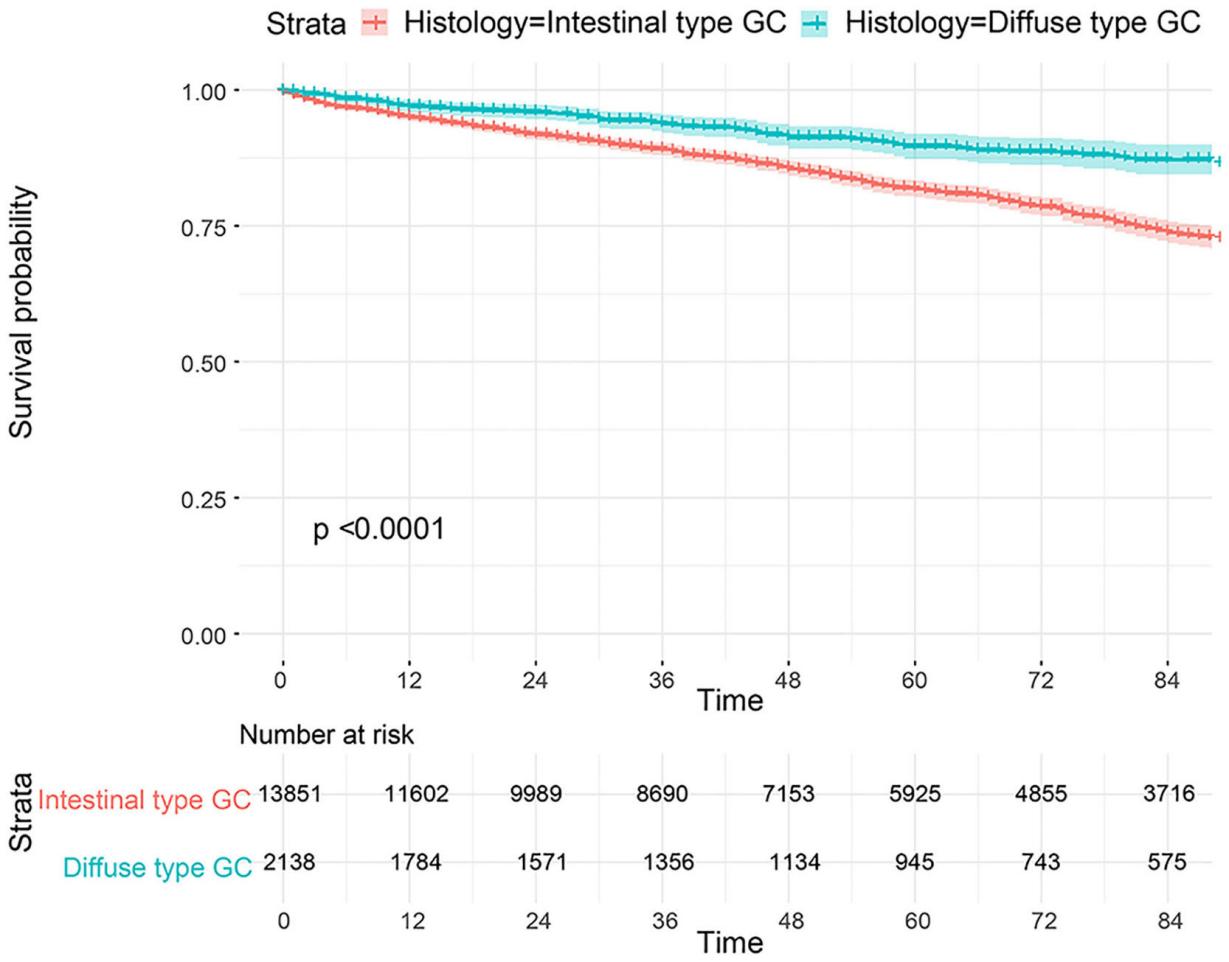
Comparison of survival for 15,989 patients from the SEER database between intestinal type and diffused type.

**Figure 5:**
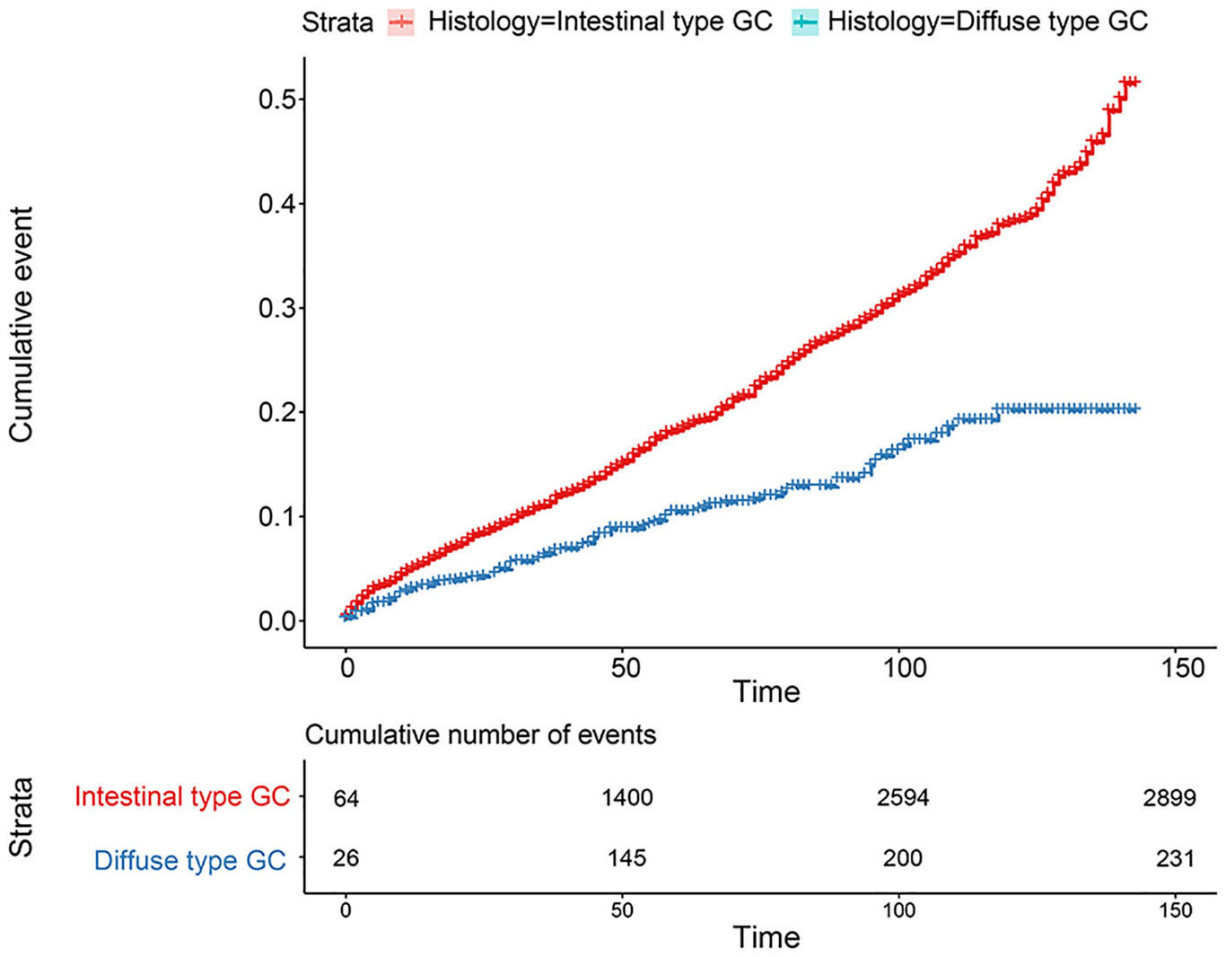
Comparison of cumulative probability for 15,989 patients from the SEER database between intestinal type and diffused type.

**Figure 6:**
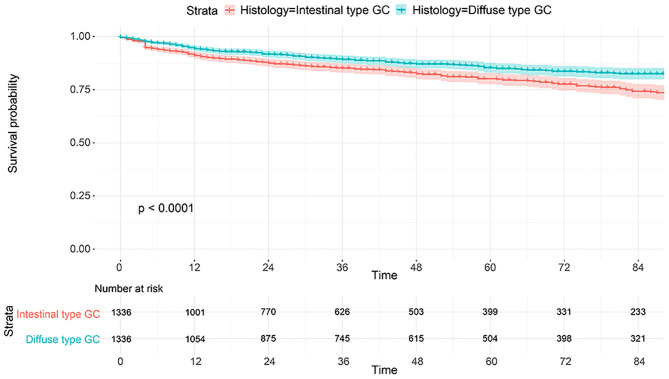
Comparison of CSS for 2,672 patients between intestinal type and diffused type after PSM.

**Figure 7:**
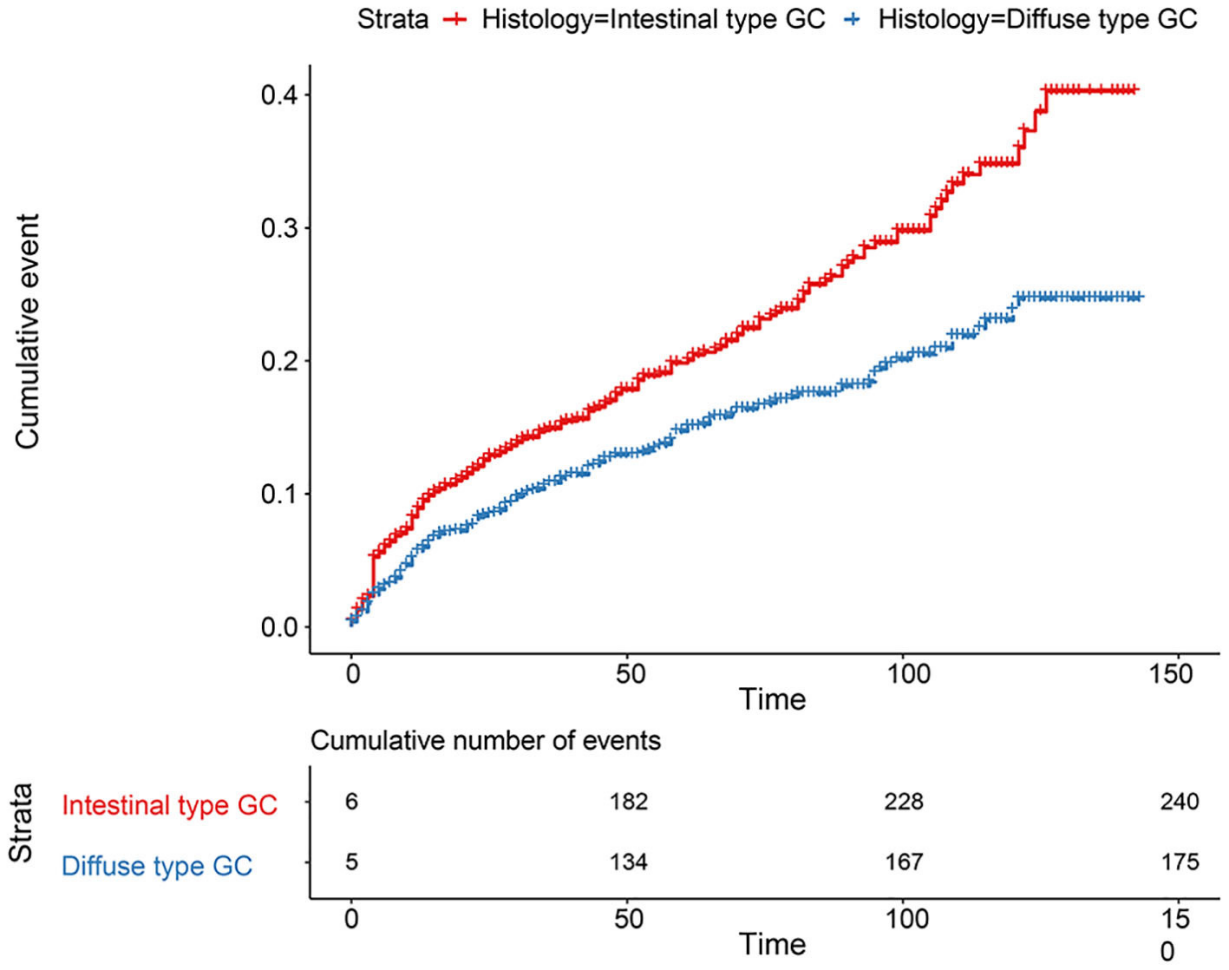
Comparison of cumulative probability for 2,672 patients between intestinal type and diffused type after PSM.

The flowchart in Figure 3 shows that 15,989 patients from the SEER database were enrolled based on the inclusion criteria. All patients were distributed into intestinal-type and diffused-type GC groups according to our predefined aims. Table 1 shows the demographic and clinical characteristics of patients in the two groups. The two groups significantly differed in age (*P* = 0.000), race (*P* = 0.000), sex (*P* = 0.000), N stage (*P* = 0.000), tumor size (*P* = 0.000), pathology grade (*P* = 0.000), regional node ratio (*P* = 0.000), and T stage and metastasis (*P* = 0.000). Patients with diffused-type GC were inclined to be younger, aged <60 years (71.52 vs. 54.8%), were of black race (12.21 vs. 5.97%), had no lymph node metastasis (45.18 vs. 24.84%), had lower lymph node-positive ratio (86.62 vs. 43.19%), were female (48.15 vs. 15.34%), had a bigger tumor size (30.03 vs. 13.43%), had advanced tumor (5.1 vs. 1.65%), and had poorly and undifferentiated carcinoma (96.21 vs. 76.41%) compared to the intestinal-type GC group. To investigate survival between the two groups, we performed a survival curve and cumulative event plot. As shown in Figures 4, 5, patients with intestinal-type GC had poorer survival than patients with diffused-type GC. Because of the imbalanced basic information, we performed PSM using R software to eliminate confounding factors. As shown in Table 2, we matched 1,366 intestinal-type GC patients with 1,366 diffused-type GC patients. All variables were balanced as demonstrated by the *P*-value (*P* > 0.05). We performed univariate Cox regression and found that histology was an independent risk factor (diffused vs. intestinal type, HR = 0.56, 95% CI: 0.45–0.78). Kaplan–Meier curves and cumulative event plotting of CSS showed that patients with intestinal-type GC had a poorer prognosis compared to diffused-type GC (Figures 6, 7).

**Table 1:** Patients' demographics, clinical characteristics at diagnosis.

**Variables**	**Total (%)**	**Intestinal type**	**Diffuse type**	***P*-Value**
*n*	15,989	13,851	2,138	
**Age**				0.000
<60	9,119 (57.03%)	7,590 (54.8%)	1,529 (71.52%)	
≥60	6,870 (42.97%)	6,261 (39.16%)	609 (28.48%)	
**Race**				0.000
White	12,835 (80.27%)	11,556 (83.43%)	1,279 (59.82%)	
Black	1,088 (6.8%)	827 (5.97%)	261 (12.21%)	
Other	2,066 (12.92%)	1,468 (10.6%)	598 (27.97%)	
**Sex**				0.000
Male	12,827 (80.22%)	11,726 (84.66%)	1,101 (51.5%)	
Female	3,162 (19.78%)	2,125 (15.34%)	1,037 (48.5%)	
**Pathology Grade**				0.000
Well/moderately differentiated	3,348 (20.94%)	3,267 (23.59%)	81 (3.79%)	
Poorly and undifferentiated	12,641 (79.06%)	10,584 (76.41%)	2,057 (96.21%)	
**T stage**				0.000
T1	2,703 (16.91%)	2,057 (14.85%)	644 (30.1%)	
T2	11,537 (72.16%)	10,609 (76.6%)	928 (43.4%)	
T3	1,402 (8.77%)	942 (6.8%)	460 (21.5%)	
T4	347 (2.17%)	241 (1.74%)	106 (5%)	
**Metastasis**				0.000
No	15,652 (97.89%)	13,623 (98.35%)	2,029 (94.9%)	
Yes	337 (2.11%)	228 (1.65%)	109 (5.1%)	
**Tumor size**				0.000
≤2 cm	2,297 (14.37%)	1,707 (12.32%)	590 (27.6%)	
≤3 cm	1,463 (9.15%)	1,081 (7.8%)	382 (17.87%)	
≤5 cm	9,727 (60.84%)	9,203 (66.44%)	524 (24.5%)	
>5 cm	2,502 (15.65%)	1,860 (13.43%)	642 (30.03%)	
**Lymphnode positive ratio**				0.000
≤0.56	7,834 (49%)	5,982 (43.19%)	1,852 (86.62%)	
>0.56	8,155 (51%)	7,869 (56.81%)	286 (13.38%)	
**N stage**				0.000
N0	4,406 (27.56%)	3,440 (24.84%)	966 (45.18%)	
N1	1,265 (7.91%)	2,298 (16.59%)	700 (32.74%)	
N2	8,328 (52.09%)	7,977 (57.59%)	351 (16.42%)	
N3	257 (1.61%)	136 (0.98%)	121 (5.66%)	

**Table 2:** Patients' demographics, clinical characteristics at diagnosis after propensity score matching for analyzing the survival between intestinal type and diffused type.

**Variables**	**Total (%)**	**Intestinal type**	**Diffuse type**	***P*-Value**
*n*	2,672	1,336	1,336	
**Age**				0.36
<60	1,803 (67.48%)	900 (67.37%)	903 (67.59%)	
≥60	869 (32.52%)	436 (32.63%)	433 (32.41%)	
**Race**				0.575
White	1,579 (59.09%)	790 (59.13%)	789 (59.06%)	
Black	285 (10.67%)	150 (11.23%)	135 (10.1%)	
Other	808 (30.24%)	396 (29.64%)	412 (30.84%)	
**Sex**				0.816
Male	1,422 (53.22%)	714 (53.44%)	708 (53%)	
Female	1,250 (46.78%)	622 (46.56%)	628 (47%)	
**Pathology grade**				0.747
Well/Moderately differentiated	97 (3.63%)	52 (3.89%)	45 (3.37%)	
Poorly and Undifferentiated	2,575 (96.37%)	1,284 (96.11%)	1,291 (96.63%)	
**Lymph node metastasis**				0.251
NO	1,388 (51.95%)	685 (51.26%)	703 (52.62%)	
N1	977 (36.56%)	509 (38.1%)	468 (35.03%)	
N2	266 (9.96%)	125 (9.36%)	141 (10.55%)	
N3	41 (1.53%)	17 (1.28%)	24 (1.8%)	
**Metastasis**				0.922
No	2,563 (95.92%)	1,282 (95.96%)	1,281 (95.88%)	
Yes	109 (4.08%)	54 (4.04%)	55 (4.12%)	
**Tumor size**				0.841
≤2cm	813 (30.42%)	409 (30.61%)	405 (30.31%)	
≤3cm	486 (18.19%)	246 (18.41%)	239 (17.89%)	
≤5cm	728 (27.25%)	357 (26.72%)	371 (27.77%)	
>5cm	645 (24.14%)	324 (24.25%)	321 (24.03%)	
**Lymphnode positive ratio**				0.235
≤0.56	2,449 (91.65%)	1,216 (91.02%)	1,233 (92.29%)	
>0.56	223 (8.35%)	120 (8.98%)	103 (7.71%)	
**T stage**				0.432
T1	929 (34.77%)	450 (33.68%)	479 (35.85%)	
T2	1,225 (45.85%)	629 (47.08%)	596 (44.61%)	
T3	448 (16.77%)	226 (16.92%)	222 (16.61%)	
T4	70 (2.61%)	31 (2.32%)	39 (2.92%)	

## Discussion

We summarized that the incidence of GC decreased in adults older than 20-year-olds from 1975 through 2015, and the incidence of regional, distant, and unstaged GC declined over time but that of local-stage GC increased. According to the Lauren classification (10), we found that the incidence increased for diffused-type GC, but the opposite trend was found for intestinal-type GC. A significantly decreased incidence was observed in patients diagnosed with intestinal-type GC in 40–49-year-olds, but the incidence of patients with diffused-type GC increased.

Our study complements previous work documenting a race-independent increase in the incidence of GC in persons lower than 50 years of age (20). The increased incidence of GC at an earlier stage was attributable to earlier detection (21, 22). For intestinal-type GC, a distinct decrease was detected from 1975 to 2015, which suggests a credible effect of eradicating Hp in preventing the occurrence of GC (23). The increasing trend of diffused-type GC, especially at an earlier age (40–49 years old), suggests that there has been a real increase in risk (22). According to some studies, obesity was apparently associated with gastrointestinal cancer and the number of obese young people was increased, which accounted for the increased incidence of younger patients with diffused-type GC (24, 25). To our knowledge, signet ring cell carcinoma which belongs to diffused-type GC was more frequent to occur in younger patients, and we also found it to be increased in young patients with diffused type (26). As for the increased younger patients, some studies found that patients were more likely to have Epstein–Barr virus and gene mutation such as CHD1 and microsatellite instability (MSI) subtype, suggesting that younger patients may have a more aggressive GC subtype (26, 27). The most well-known feature was the high frequency of CHD1 alteration, inducing the GC histology type of younger patients to be diffused (28, 29). In addition, diffused-type GC was found to be more frequently associated with mutations of CDH1 and p53 and amplification of Her2 (13), leading to higher malignancy compared to the intestinal type. The reasons why diffused types were inclined to be black and female patients remained unknown, of which special genomic alteration would be one plausible cause.

As for the Lauren classification proposed in 1965 (10), there is limited evidence to demonstrate its clinical significance. Some previous studies suggested that patients with intestinal-type GC had a better prognosis than patients with diffused-type GC (30–34). Some studies considered that the Lauren's grade distribution did not change with the progression from an early to advanced gastric carcinoma (35), and Lauren classification was not an independent prognostic factor (16, 36). However, some studies showed that patients with diffused-type GC had a similar or better prognosis than patients with intestinal-type GC (37, 38), which is consistent with our results. Our study performed PSM to adjust for other risk factors and found that patients with intestinal-type GC had poorer survival. The explanation to our results were manifold such as the different recurrent and metastatic rates. Some studies found that the genomic profile between intestinal type and diffused type was different; for instance, intestinal-type GC tended to have chromosomal instability, which was associated with poor prognosis while diffused type preferred to be genomically stable (39). Also, considering the recurrent rate, patients with intestinal-type GC were higher than those who had diffused-type GC (54.4 vs. 37.4%, *P* < 0.001) (34). Additionally, patients with intestinal type were more associated with distant metastasis (34). The reasons for these contradictory results may be due to the clinical heterogeneity and different sample size. Therefore, more retrospective studies with a larger sample size are needed to perform to validate the findings.

The limitations in our study should not be neglected. First, our study did not investigate the correlation between other factors and CSS. Second, some factors, such as the depth of invasion and lymphatic vessel involvement, were not included in our analyses because of the limited information provided in the SEER database. Patients with unavailable information could affect our results to some extent. Therefore, further studies should be performed to examine whether the histology type may be used as an independent predictor for prognosis after adjusting for more confounding factors.

It could be concluded that GC incidence decreased by analyzing the SEER database from 1975 through 2015. The incidence of diffused-type GC in 40–49-year-old patients was increased, but total GC and intestinal-type GC incidence decreased, which warrants further research to elucidate the underlying causes. As for the prognosis between the two types, patients with intestinal-type GC had a poorer prognosis than those with diffused-type GC in our PSM analysis by the SEER database; however, it needs more retrospective studies to investigate because of the clinical heterogeneity. As far as clinicians are concerned, it would be better to treat intestinal-type or diffused-type GC with different altitudes, especially for younger patients with GC. Diffused-type GC should be attached with greater attention in younger patients. Our data revealed that survival of patients with diffused or intestinal type should be assessed.

## Data Availability Statement

Publicly available datasets were analyzed in this study. This data can be found here: https://seer.cancer.gov/data/.

## Author Contributions

C-TT: data collection, data analysis, and manuscript writing. LZ and JY: data analysis. CZ and YC: project development. All authors contributed to the article and approved the submitted version.

## Conflict of Interest

The authors declare that the research was conducted in the absence of any commercial or financial relationships that could be construed as a potential conflict of interest.
